# Respiratory virus disease and outcomes at a large academic medical center in the United States: a retrospective observational study of the early 2023/2024 respiratory viral season

**DOI:** 10.1128/spectrum.01116-24

**Published:** 2024-08-20

**Authors:** Heba H. Mostafa, Amary Fall, Julie M. Norton, Jaiprasath Sachithanandham, Madeline Yunker, Omar Abdullah, Ann Hanlon, Linda Gluck, C. Paul Morris, Andrew Pekosz, Eili Y. Klein

**Affiliations:** 1 Department of Pathology, Division of Medical Microbiology, Johns Hopkins School of Medicine, Baltimore, Maryland, USA; 2 W. Harry Feinstone Department of Molecular Microbiology and Immunology, The Johns Hopkins Bloomberg School of Public Health, Baltimore, Maryland, USA; 3 Integrated Research Facility, Division of Clinical Research, National Institute of Allergy and Infectious Diseases, National Institutes of Health, Frederick, Maryland, USA; 4 Department of Emergency Medicine, Johns Hopkins School of Medicine, Baltimore, Maryland, USA; 5 Center for Disease Dynamics, Economics, and Policy, Washington, DC, USA; Quest Diagnostics, Secaucus, New Jersey, USA

**Keywords:** influenza, SARS-CoV-2, respiratory syncytial virus, enterovirus, rhinovirus, genome analysis

## Abstract

Respiratory disease, attributed to influenza, respiratory syncytial virus (RSV), and SARS-CoV-2, was reported nationally during the 2023/2024 respiratory viral season. The emergence of novel SARS-CoV-2 variants was considered a significant factor contributing to the rise in COVID-19 cases. Data from the Johns Hopkins Hospital System (JHHS) showed that enterovirus/rhinovirus had also been circulating at high rates. Analyzing clinical outcomes of the most prevalent respiratory viruses is crucial for understanding the role of circulating viral genotypes. A retrospective cohort of patients who tested positive for SARS-CoV-2, influenza, RSV, or enterovirus/rhinovirus between 1 June and 31 December 2023 was included in the study. Remnant clinical samples were utilized for targeted viral whole-genome sequencing and genotyping. Patients’ metadata and outcomes following infection were studied, stratified by viral variants and genotypes. The increase of SARS-CoV-2 positivity in December was associated with the predominance of JN.1. Admissions for patients under 18 years old were primarily associated with enterovirus/rhinovirus and RSV, while older age groups were mainly linked to SARS-CoV-2 and influenza infections. SARS-CoV-2-related admissions increased with the predominance of the JN.1 variant in December. No significant difference in admissions for influenza subtypes, rhinovirus species, or SARS-CoV-2 variants was observed. RSV A was associated with slightly higher odds of admission compared with RSV B. Our data highlight the importance of systematically analyzing respiratory viral infections to inform public health strategies and clinical management, especially as SARS-CoV-2 becomes endemic. The findings highlight the value of expanded genomic surveillance in elucidating the clinical significance of viral evolution.

**IMPORTANCE:**

The analysis of the epidemiology and clinical outcomes of multiple co-circulating respiratory viruses in the early 2023/2024 respiratory virus season highlights the emergence of the SARS-CoV-2 JN.1 variant as well as underscores the importance of enterovirus/rhinovirus in respiratory infections. Understanding these dynamics is essential for refining public health strategies and clinical management, especially as SARS-CoV-2 transitions to an endemic status. This work emphasizes the need for ongoing surveillance, robust diagnostic algorithms, and detailed genomic analyses to anticipate and mitigate the burden of respiratory viral infections, ultimately contributing to more informed decision-making in healthcare settings and better patient outcomes.

## INTRODUCTION

The burden of circulating respiratory viruses can vary from season to season, influenced by factors such as virus genotypes and the susceptibility of exposed populations. The 2023/2024 respiratory season in the United States (US) saw the circulation of influenza, respiratory syncytial virus (RSV), and SARS-CoV-2, leading to a nationwide surge in hospital admissions and mortality (1). The emergence and circulation of the SARS-CoV-2 JN.1 variant correlated with spikes in RSV and influenza, contributing to the increased COVID-19 cases (2). In the 2023/2024 season, seasonal influenza viruses predominately included H1N1, with co-circulation of H3N2 and influenza B, which contrasts with the prior two seasons dominated by H3N2 (3, 4). As SARS-CoV-2 transitions into an endemic status, understanding the outcomes of infections with this virus compared with other highly prevalent respiratory viruses, co-infections, and the impact of circulating genotypes and variants becomes crucial (5). However, the algorithms used in laboratory diagnosis of respiratory pathogens may challenge a clear understanding of the association of infrequently screened viruses with disease severity. While laboratory diagnosis of symptomatic viral respiratory tract infections currently integrates SARS-CoV-2 with influenza and RSV, additional respiratory pathogen testing is notably less frequent and largely restricted to immunocompromised, hospitalized, and, to a lesser extent, elderly and pediatric patients (6). Furthermore, data comparing the clinical presentations and outcomes of respiratory viral infections for the 2023/2024 season, factoring in viral variants and genotypes, are not widely reported. We utilized the comprehensive Johns Hopkins Hospital System (JHHS) respiratory viral testing data to evaluate the relative circulation of respiratory viruses and demonstrated that the highest positivity rate was for enterovirus/rhinovirus, followed by influenza, SARS-CoV-2, and RSV. We then characterized the circulating genotypes for these four groups of viruses from 1 June to 31 December 2023, using whole genome sequencing or genotyping, and analyzed the likelihood of severe outcomes for patients infected with each virus group/viral genotype.

## MATERIALS AND METHODS

### Patient data and clinical samples

This study is a retrospective observational cohort study that used remnant nasopharyngeal/nasal swab samples after standard of care testing (from 1 June 2023 to 31 December 2023) for SARS-CoV-2, influenza, RSV, and enterovirus/rhinovirus. At JHHS, testing is performed for inpatients and outpatients (five acute care hospitals and a large network of ambulatory care practices). Clinical testing was performed using the NeuMoDx SARS-CoV-2 (Qiagen), Xpert Xpress SARS-CoV-2/Flu/RSV (Cepheid), and the ePlex Respiratory Pathogen Panel 2 (Roche) (7–9). Patients’ clinical and demographic data were extracted in bulk from the electronic medical record system.

### Sample size

Between 1 June 2023 and 31 December 2023, 64,848 samples were tested for SARS-CoV-2, 37,571 samples were tested for influenza and RSV, and 8,980 samples were tested for enterovirus/rhinovirus [in addition to other respiratory viruses that include endemic coronaviruses, human parainfluenza viruses, and adenovirus (Fig. S1)]. Of those samples, 1,542 of 37,571 (4.1%) were positive for influenza A, 254 of 37,571 (0.68%) were positive for influenza B, 2,072 of 37,571 (5.5%) were positive for RSV, 3,963 of 64,848 (6.1%) were positive for SARS-CoV-2, and 1,258 of 8,980 (14%) were positive for enterovirus/rhinovirus. Positive samples were randomly selected for whole viral genome sequencing or viral genotyping, which was attempted for 526 (29.3%) influenza, 428 (20.7%) RSV, 1,673 (42.2%) SARS-CoV-2, and 829 (75.9%) enterovirus/rhinovirus specimens. Complete genomes and genotypes and associated clinical data were available for 330 (62.7%) influenza, 392 (91.6%) RSV, 1,129 (67.5%) SARS-CoV-2, and 677 (81.7%) enterovirus/rhinovirus-infected unique patients.

### Viral complete genome sequencing and genotyping

Nucleic acid extraction was performed using the chemagic viral RNA/DNA kit and the Chemagic 360 system (PerkinElmer) according to the manufacturer’s specifications. Influenza and SARS-CoV-2 whole genome sequencing and analysis were performed as described previously (3, 10). Enterovirus/rhinovirus genotyping was performed by targeted sequencing of the VP4/VP2 genomic region (11). Amplification was performed using the LunaScript RT SuperMix Kit (NEB, New England), according to the manufacturer’s instructions. Primers used for amplification were as follows: F484: 5′ CGGCCCCTGAATGYGGCTAA 3′, R1126: 5′ ATCHGGHARYTTCCAMCACCA 3′, and REV-R2: 5′'GTCGGGGARYTTCCAMTACCA 3′. PCR was carried out in a 25-µL reaction containing 5 µL of cDNA, 12.5 µL of Q5 Hot Start High-Fidelity 2× Master Mix (New England Biolabs), 1 µL of each primer (diluted at 10 µM), and 5.5 µL nuclease-free water. Thermocycling steps included 98°C for 30 s, 35 cycles of 95°C for 30 s, 58°C for 40 s, and 65°C for 1 min, with a final extension at 65°C for 5 min. Library prep was performed using the native barcoding kit (EXP-NBD196) and the NEBNext ARTIC Library Prep Kit according to the manufacturer’s instructions, and sequencing was performed using R9.4.1 flow cells on a GridION (Oxford Nanopore Technologies). Fastq files were analyzed using an in-house pipeline with steps that included analysis against a database consisting of all enterovirus/rhinovirus reference genomes, selecting the closest reference, running mini_assemble within pomoxis to generate a draft genome, using medaka consensus to further enhance the draft genome and establish a consensus sequence, and finally, evaluating depth with samtools. The RIVM genotyping tool was used for species and type assignment (12). RSV-positive samples were differentiated into RSV A and RSV B groups using a real-time PCR assay. The Luna Probe One-Step RT-qPCR (New England Biolabs) was used following the manufacturer’s instructions. Primers and probes included the following: for RSV A: RSV-A N792 771F: 5′-TGCTAAGACYCCCCACCGTAAC-3′, RSV-A N723 749R: 5′-GGATTTTTGCAGGATTGTTTATGH-3′, RSV-A N768 753P (probe): 5′-/56-HEX/C + ACT + TGC + CCT + G + CW + CC/A131ABkFQ-3′, for RSV B: RSV-B N749 726F: 5′-GCATTCATAAACAATCCTGCAAAG-3′, RSV-B N680 699R: 5′-GGCAATGCACAATCATCCAC-3′, and RSV-B N722 702P (probe): 5′-/56-FAM/CTTCAACTC/ZEN/TAC/TRCCCCCTC/31ABkFQ. Whole genome sequencing was used to determine the genotypes following a targeted nested PCR amplification approach as previously described (13). Twenty primer pairs for RSV A or RSV B were used for the initial amplification of RSV A/B samples, generating 20,900 base pair amplicons. A shortened extension time, 1 min 15 s, was used. Two microliters of first-round PCR products was used for targeted 20-amplicon nested PCR. PCR products were used for the library prep using the NEBNext ARTIC Library Prep Kit and the Native Barcoding Kit 96 V14 (SQK-NBD114.96) according to the manufacturer’s instructions. Sequencing was performed with R10.4.1 flow cells (FLO-PRO114M) on the PromethION 2 solo device (Oxford Nanopore Technologies). Analysis was performed with an in-house pipeline as described for enterovirus/rhinovirus. Genotypes were assigned using Nexclade (https://clades.nextstrain.org) and phylogenetic analysis.

### Cell culture

Recovery of infectious SARS-CoV-2 in VeroE6-ACE2-TMPRSS2 was previously described (14). Samples with high-quality genomes (average depth of 400 and average coverage > 95%) were selected for cell culture to control for relative viral load.

### Statistical analysis

Multivariable logistic regression was used to evaluate the odds ratio of admission and need for supplemental oxygen. Analyses were done separately for each pathogen stratified by subclade/variant/species type, controlling for age, gender, comorbidities, smoking, pregnancy, emergency department visit, and co-infections. All statistical analyses were conducted in Stata 17 (StataCorp LP, College Station, TX).

### Role of the funding source

The funder of the study had no role in the study design, data collection, data analysis, data interpretation, or writing of the report.

## RESULTS

### Respiratory viral positivity, influenza-like illness, and admissions

**Fig 1:**
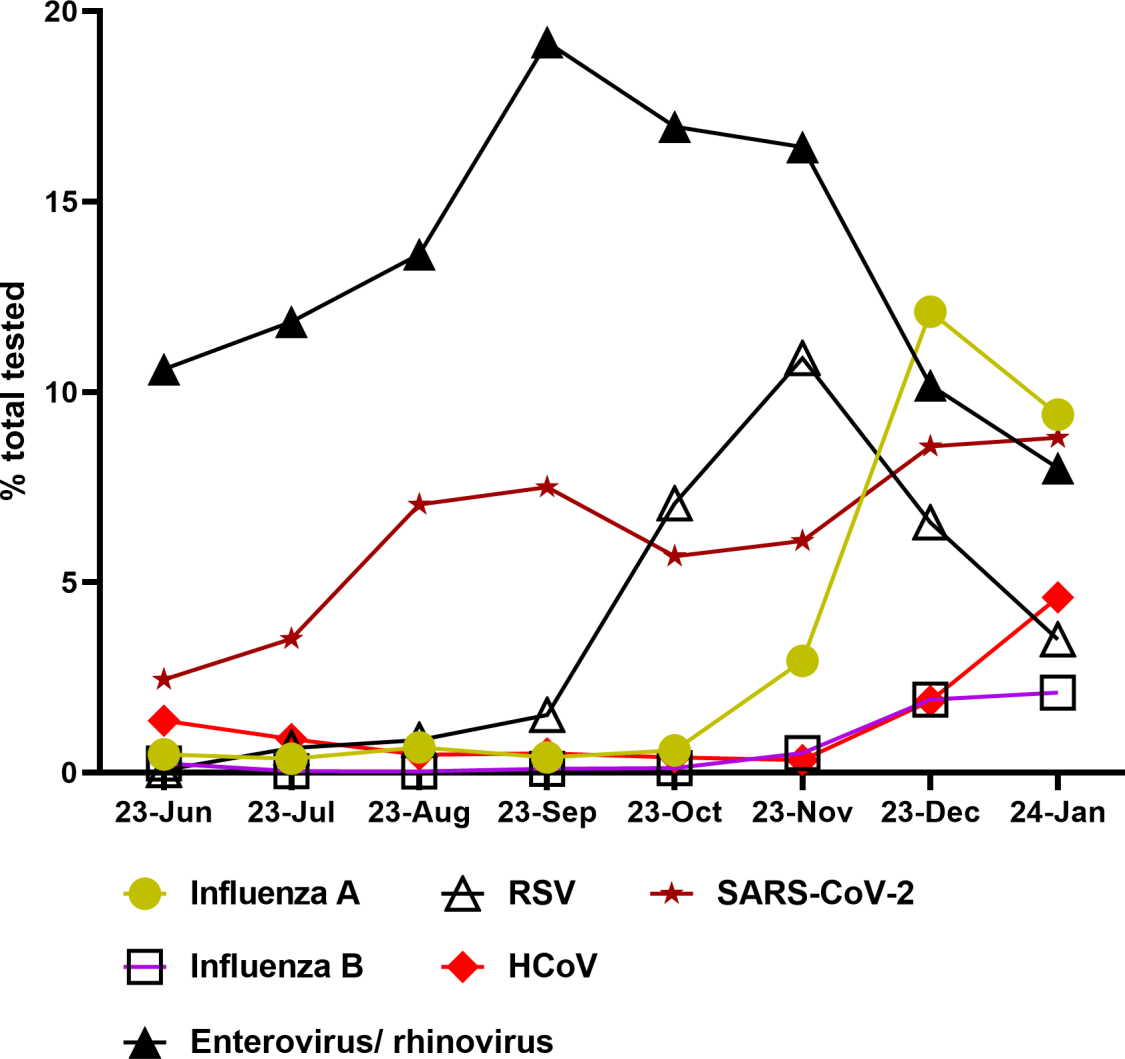
Positivity rates of respiratory virus testing at JHHS from June 2023 to December 2023. Data up to 21 January 2024 were included for context. HCoV, human endemic coronaviruses.

Of the tested respiratory viruses at JHHS, SARS-CoV-2, influenza, RSV, and enterovirus/rhinovirus showed the highest positivity rates. Enterovirus/rhinovirus had the highest positivity, starting at 10.6% in June and peaking at 19.2% in September. RSV positivity peaked at 10.9% in November, followed by a peak in influenza A positivity in December, which reached 12.1%. The SARS-CoV-2 positivity rate increased in August and September to 7% and 7.5%, respectively, followed by a slight reduction to 5.7% in October, and then an increase in November and December to 6.1% and 8.6% (Fig. 1). Notably, the trend of increased SARS-CoV-2 positivity in December was associated with a similar trend of increased endemic coronavirus positivity (Fig. 1; Fig. S1).

**Fig 2:**
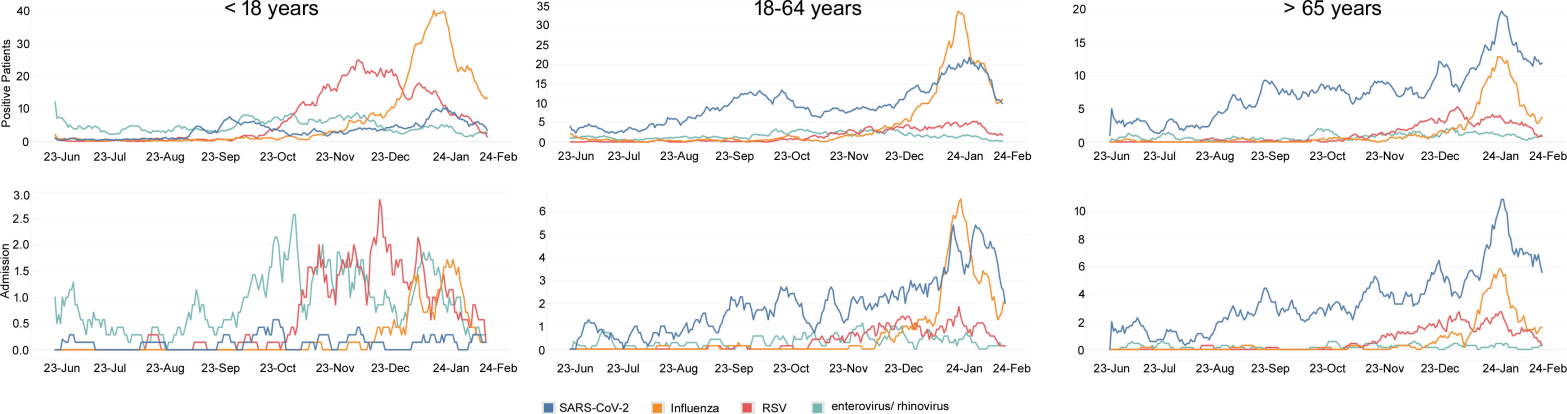
Encounters of respiratory virus infections and admissions at JHHS from June 2023 to December 2023. Data shown as 7 days rolling average. Data up to 21 January 2024 were included for context.

The vast majority of RSV infections were in patients younger than 18 years (Table 1; Fig. 2, detailed age groups are shown in Table S3). For influenza and enterovirus/rhinovirus, most infections were also in patients younger than 18. However, for SAR-CoV-2, the age distribution of infections was nearly flat (Table 1), and age was strongly associated with increased admission likelihood (Table S1).

Enterovirus/rhinovirus-infected patients younger than 18 had higher admission rates compared with the other viral pathogens (Fig. 2; Table 1). Patients with positive tests for more than one respiratory virus accounted for only 2%–3% of all patients tested (Table 1). The vast majority of asymptomatic patients in our cohort were SARS-CoV-2 positive (Table 1).

**TABLE 1:** Patient demographics

	Enterovirus/rhinovirus	RSV	Influenza	SARS-CoV-2
*N*	677	392	330	1,091
Female	319 (47.1%)	202 (51.5%)	169 (51.2%)	589 (54.0%)
Age				
0–17	370 (54.7%)	313 (79.9%)	184 (55.8%)	235 (21.5%)
18–44	111 (16.4%)	29 (7.4%)	69 (20.9%)	260 (23.8%)
45–64	95 (14.0%)	23 (5.9%)	41 (12.4%)	232 (21.3%)
65–79	78 (11.5%)	24 (6.1%)	28 (8.5%)	230 (21.1%)
80+	23 (3.4%)	3 (0.8%)	8 (2.4%)	134 (12.3%)
Comorbidities				
Atrial fibrillation	64 (9.5%)	21 (5.4%)	23 (7.0%)	165 (15.1%)
Cancer	292 (43.1%)	93 (23.7%)	99 (30.0%)	544 (49.9%)
Cerebrovascular disease	111 (16.4%)	22 (5.6%)	28 (8.5%)	241 (22.1%)
Coronary artery disease	193 (28.5%)	48 (12.2%)	61 (18.5%)	401 (36.8%)
Diabetes	115 (17.0%)	29 (7.4%)	33 (10.0%)	288 (26.4%)
Heart failure	104 (15.4%)	27 (6.9%)	31 (9.4%)	210 (19.2%)
Hypertension	277 (40.9%)	68 (17.3%)	75 (22.7%)	573 (52.5%)
Immunosuppression	335 (49.5%)	81 (20.7%)	66 (20.0%)	395 (36.2%)
Kidney disease	205 (30.3%)	50 (12.8%)	47 (14.2%)	340 (31.2%)
Lung disease	286 (42.2%)	115 (29.3%)	104 (31.5%)	348 (31.9%)
Smoker	105 (15.5%)	19 (4.8%)	44 (13.3%)	189 (17.3%)
Pregnant	19 (2.8%)	5 (1.3%)	7 (2.1%)	26 (2.4%)
Coinfection	15 (2.2%)	12 (3.1%)	10 (3.0%)	23 (2.1%)
ED visit	482 (71.2%)	346 (88.3%)	304 (92.1%)	941 (86.3%)
Admitted	469 (69.3%)	123 (31.4%)	85 (25.8%)	516 (47.3%)
0–17	237 (64.1%)	77 (24.6%)	17 (9.2%)	39 (16.6%)
18–44	74 (66.7%)	11 (37.9%)	20 (29.0%)	77 (29.6%)
45–64	80 (84.2%)	16 (69.6%)	20 (48.8%)	116 (50.0%)
65–79	58 (74.4%)	16 (66.7%)	22 (78.6%)	168 (73.0%)
80+	20 (87.0%)	3 (100.0%)	6 (75.0%)	116 (86.6%)
Symptomatic at admission^*a*^	462 (68.2%)	109 (27.8%)	80 (24.2%)	359 (32.9%)
ICU-level care	140 (20.7%)	32 (8.2%)	13 (3.9%)	75 (6.9%)
Supplemental O_2_	295 (43.6%)	95 (24.2%)	55 (16.7%)	287 (26.3%)

^*a*^ Patients had to have a chief complaint suggestive of respiratory infection or a clinical impression of an upper respiratory infection and not be coinfected.

### Circulating respiratory viral variants

**Fig 3:**
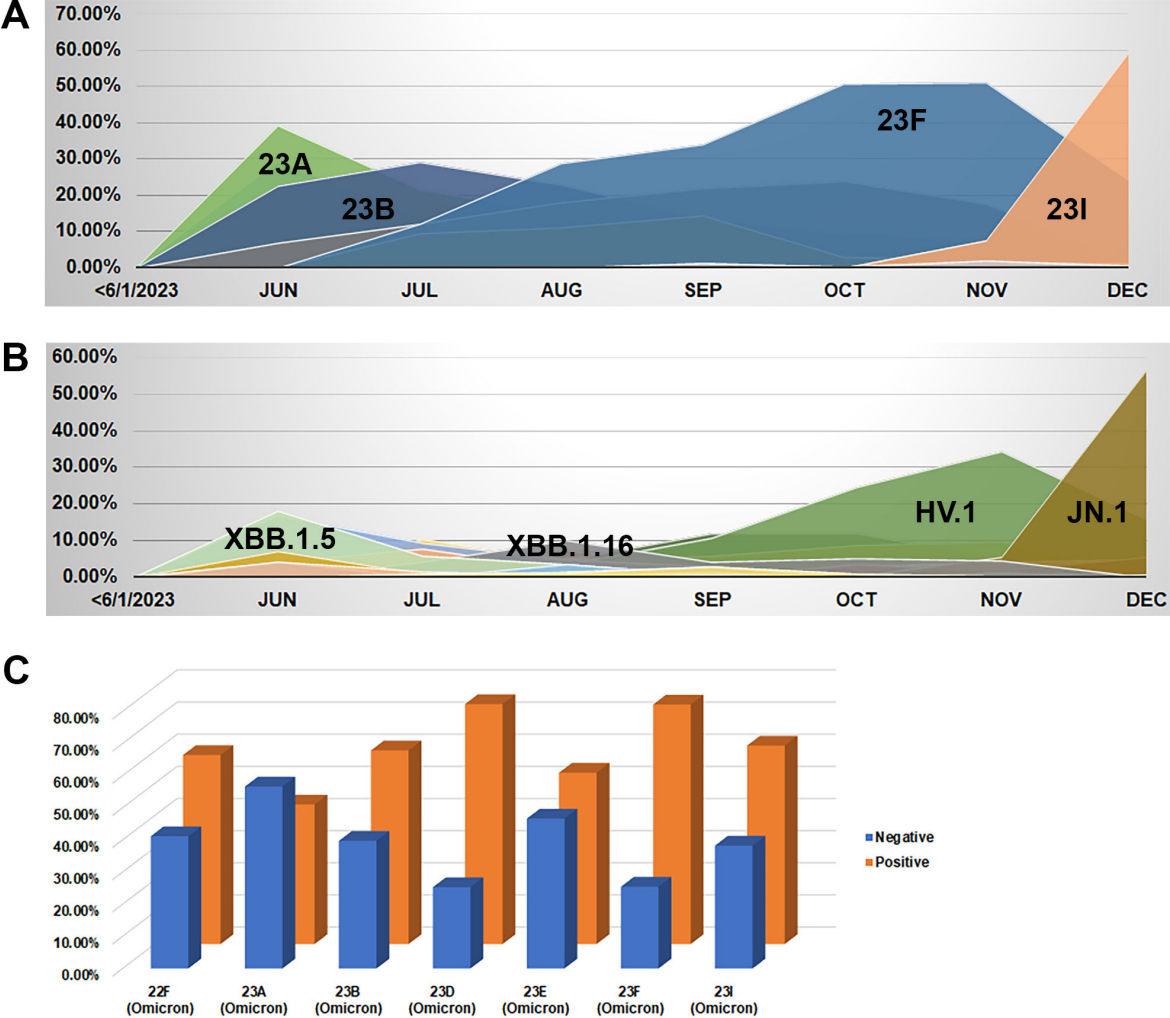
SARS-CoV-2 circulating clades (**A**) and lineages (**B**) at JHHS from June to December 2023 and percentage recovery of SARS-CoV-2 from positive clinical samples in cell culture (**C**).

Several Omicron clades and lineages circulated between June and December 2023 (Fig. 3; Table 2; Tables S3, and S4). A predominance of clade 23I, specifically variant JN.1, was notable in December 2023, following the predominance of clade 23F, variant HV.1, in the prior 2 months (Fig. 3A and B). For detailed lineage distribution per clade, refer to Tables S4 and S5. When the recovery of JN.1 in cell culture was compared with prior lineages, no significant difference was observed (Fig. 3C ; Table S6). Notably, the predominance of JN.1 correlated with the increase in influenza-like illness (ILI) and admissions in patients older than 18 years in December 2023 (Fig. 2).

**TABLE 2:** Species/groups/subclades/clades identified in our study cohort (detailed in Tables S4 through S9)

Enterovirus/rhinovirus	
Enterovirus A/B/C/D	34 (5.0%)
Rhinovirus A	260 (38.4%)
Rhinovirus B	135 (19.9%)
Rhinovirus C	248 (36.6%)
RSV	
A	90 (23.0%)
A/B	10 (2.6%)
B	292 (74.5%)
Influenza	
H1N1	246 (74.6%)
H3N2	46 (13.9%)
Influenza B	38 (11.5%)
SARS-CoV-2	
22F (Omicron)	54 (5.0%)
23A (Omicron)	144 (13.2%)
23B (Omicron)	154 (14.1%)
23D (Omicron)	171 (15.7%)
23E (Omicron)	71 (6.5%)
23F (Omicron)	370 (33.9%)
23I (Omicron)	101 (9.3%)
Other	26 (2.4%)

During the timeline of this study, influenza H1N1 predominated (Table 2) with only clades 6B.1A.5a.2a.1 and 6B.1A.5a.2a being detected (Table S7). In the limited influenza H3N2 and influenza B samples, the 3C.2a1b.2a.2a.3a.1 clade and the V1A.3a.2 clade (Table S7) predominated, respectively.

Enterovirus/rhinovirus species circulating were primarily rhinovirus A, followed by rhinoviruses C and B, with low detection of enteroviruses, mainly enterovirus B, and minimal circulation of enterovirus D during this time frame (Table 2; Table S8). RSV group B GB5.0.5a genotype predominated, followed by RSV A genotype GA2 (Table 2; Table S9).

Analysis of the effect of viral variant, genotype, or species on admission rates showed only a limited statistically significant difference. Specifically, RSV A was associated with a slightly higher odds of admission, but no increase in the odds of needing supplemental oxygen. Rhinovirus was associated with an increased probability of admission and need for supplemental oxygen compared with enterovirus, though the number of enteroviruses was low and there were no differences noted between rhinovirus species. For the other pathogens, we found no statistically significant difference in admission rates or need for supplemental oxygen. This includes the recent SARS-CoV-2 JN.1 lineage (clade 23I), which had no statistically significant difference in the odds of admission compared with prior lineages (Table 3; Table S4).

**TABLE 3:** Odds ratios for admission and supplemental oxygen for viral groups/subclades/species/clades^*a*^

	Admission	Supplemental oxygen
RSV		
B	Reference	
A	2.0 (1.1–3.6)	1.5 (0.9–2.8)
A/B	4.5 (1.1–18.4)	2.4 (0.6–9.4)
Influenza		
H1N1	Reference	
H3N2	0.4 (0.1–1.3)	0.3 (0.1–0.9)
IVB	0.4 (0.1–1.8)	1.0 (0.3–4.2)
Enterovirus/rhinovirus		
Enterovirus	Reference	
Rhinovirus A	2.3 (1.1–5.0)	5.4 (1.8–16.3)
Rhinovirus B	2.8 (1.2–6.3)	4.0 (1.3–12.4)
Rhinovirus C	2.6 (1.2–5.5)	4.7 (1.6–14.2)
SARS-CoV-2		
22F (Omicron)	Reference	
23A (Omicron)	0.9 (0.4–2.2)	1.8 (0.8–4.4)
23B (Omicron)	1.4 (0.6–3.3)	1.5 (0.6–3.7)
23D (Omicron)	1.3 (0.6–3.0)	1.5 (0.6–3.6)
23E (Omicron)	1.2 (0.5–3.0)	0.8 (0.3–2.2)
23F (Omicron)	1.4 (0.6–3.0)	1.4 (0.6–3.2)
23I (Omicron)	1.5 (0.6–3.5)	0.9 (0.4–2.6)
Other	2.0 (0.6–6.5)	2.7 (0.8–9.5)

^*a*^ Results are part of multivariable regression controlling for sex, age, and comorbidities. See Tables S1 and S2 for other columns.

## DISCUSSION

A potential “tripledemic” due to the co-circulation of influenza, RSV, and SARS-CoV-2 during the 2023/2024 respiratory viral season was a national concern. Respiratory virus disease attributed to these viruses was notable. However, the JHHS respiratory viral testing data showed that enterovirus/rhinovirus has also been co-circulating at high rates. Concurrently, added concern related to the evolution of the SARS-CoV-2 JN.1 variant raised questions about its contribution to the severity of infections and hospital admissions.

Thus, it becomes important to systematically analyze the clinical outcomes of the most predominant respiratory viruses and to understand whether there are differences in severity associated with evolving variants. Previous studies were limited and did not consider viral variants or genotypes. For instance, a study from Brazil demonstrated that SARS-CoV-2 had the highest mortality rate among hospitalized children and adolescents diagnosed between February 2020 and February 2023, compared with other respiratory viruses (15). In Sweden, pediatric cases diagnosed between 1 August 2021 and 15 September 2022 had higher odds of admission for RSV compared with SARS-CoV-2 and influenza (16). Furthermore, a study from the Netherlands, which compared mortality associated with infections of various respiratory viruses between 1 July 2017 and 1 March 2022, revealed that mortality associated with SARS-CoV-2 was higher, especially between March and August 2020 (17).

The diagnostic algorithm for respiratory viruses challenges a clear understanding of infection outcomes when retrospectively analyzing data. Since the onset of the COVID-19 pandemic, SARS-CoV-2 was the only respiratory virus screened for in asymptomatic patients, making it the most frequently tested respiratory virus pathogen (18). Symptomatic patients during the influenza season are typically tested for influenza, SARS-CoV-2, and/or RSV, and if negative, further testing is unlikely. Extended respiratory panels are primarily considered for immunocompromised, hospitalized, and pediatric patients (19). While this algorithm may currently be the most relevant for patient management, interpreting the outcomes of infections with less commonly tested respiratory viruses poses challenges due to limited testing in specific symptomatic patient populations. Additionally, individuals with mild upper respiratory tract infections typically do not seek medical advice or get tested. These limitations introduce significant bias, and as a result, we were unable to compare infection outcomes among the four groups of viruses in our retrospective cohort. Therefore, our study was confined to describing outcomes for each viral group individually.

The seasonality of respiratory viral infections significantly influences positivity rates across different months and from year to year (20). Interestingly, the uptick in SARS-CoV-2 positivity observed in December 2023 and January 2024 in our system coincided with a rise in the positivity of endemic coronaviruses. This observation suggests that the increase in SARS-CoV-2 infections may not solely be attributed to the evolution of the JN.1 variant but could also be influenced by seasonal patterns of the virus. Notably, the trends in SARS-CoV-2 positivity in 2023 mirrored those of 2022 in our system (Fig. S1). Similarly, the trends in 2021 were comparable, with the emergence of Omicron resulting in significantly elevated positivity rates in December 2021 and January 2022. We therefore hypothesize that the rise in SARS-CoV-2 positivity during December and January months may be partly attributed to seasonal patterns as the virus transitions to an endemic state.

The seasonality of influenza and RSV viruses is well understood and extensively documented. The circulation of various influenza subtypes and clades fluctuates each season, impacting the composition of seasonal influenza vaccines annually (21). RSV seasonality exhibits alternating patterns of RSV group A predominance and seasons with both RSV groups A and B circulating (22, 23). In our system, the enterovirus/rhinovirus peak in September 2023 was predominated by rhinoviruses A and C, contrasting with that in September 2022 and 2023, which showed increased enterovirus D68 (24, 25). Changes in circulating viral variants and types from season to season can influence disease severity and outcomes. Therefore, understanding the associations between viral evolution and infection outcomes each season becomes crucial.

In addition to the testing bias mentioned earlier, our study has several limitations. The retrospective nature of the study restricted the collection of data on previous respiratory viral infections and vaccinations. Admissions among immunocompromised patients and individuals with underlying conditions can be multifactorial. Despite employing stringent criteria to define admissions associated with each virus, other factors may have influenced clinical decisions regarding hospitalization. Prospective surveillance and transmission studies for all respiratory viruses have become a national priority. These studies will provide detailed insights into the outcomes of each viral infection.

## Data Availability

Complete viral genomes that met the quality cut-offs are publicly available in GISAID Table S10.

## SUPPLEMENTAL MATERIAL

Fig. S1 spectrum.01116-24-s0001.tif Positivity rates of respiratory virus testing at JHHS from June to December 2023.

Supplemental tables spectrum.01116-24-s0002.docx Tables S1 to S9.

Table S10 spectrum.01116-24-s0003.xlsx GISAID ID numbers of submitted complete genomes used for the study.

ASM does not own the copyrights to Supplemental Material that may be linked to, or accessed through, an article. The authors have granted ASM a non-exclusive, world-wide license to publish the Supplemental Material files. Please contact the corresponding author directly for reuse.
