# Supplementary figures and images for: Respiratory virus disease and outcomes at a large academic medical center in the United States: a retrospective observational study of the early 2023/2024 respiratory viral season
Source: Microbiol Spectr. 2024 Aug 20;12(10):e01116-24. doi: 10.1128/spectrum.01116-24 (PMC11448398; doi:10.1128/spectrum.01116-24)

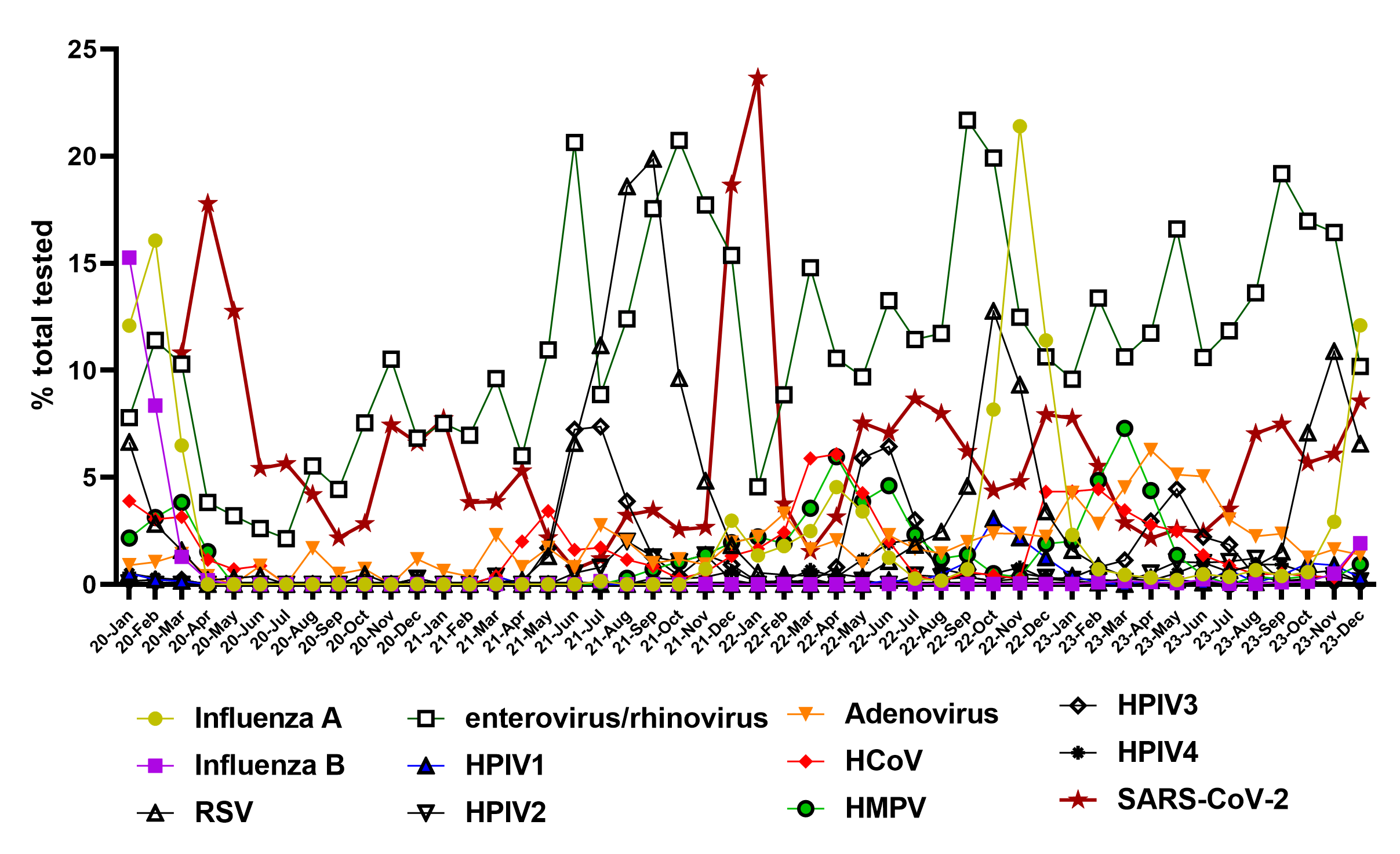

Supplement: Fig. S1 — Positivity rates of respiratory virus testing at JHHS from June to December 2023. [file spectrum.01116-24-s0001.tif]
