# Supplementary material for: Respiratory virus disease and outcomes at a large academic medical center in the United States: a retrospective observational study of the early 2023/2024 respiratory viral season
Source: Microbiol Spectr. 2024 Aug 20;12(10):e01116-24. doi: 10.1128/spectrum.01116-24 (PMC11448398; doi:10.1128/spectrum.01116-24)
Supplement: Supplemental tables — Tables S1 to S9. [file spectrum.01116-24-s0002.docx]

| Supplementary Table 1: Odds ratios for multivariable logistic regression covariates for admission | | | | |
| --- | --- | --- | --- | --- |
|  | Enterovirus/ rhinovirus | RSV | Influenza | SARS-CoV-2 |
| Female | 1.0 (0.7-1.5) | 1.0 (0.6-1.7) | 1.0 (0.5-2.2) | 0.8 (0.6-1.2) |
| Age |  |  |  |  |
| 0-17 | Reference | Reference | Reference | Reference |
| 18-44 | 1.1 (0.6-2.0) | 0.6 (0.2-2.1) | 1.2 (0.4-3.3) | 1.3 (0.8-2.2) |
| 45-64 | 1.7 (0.8-3.8) | 0.5 (0.1-3.7) | 4.4 (1.4-13.7) | 2.0 (1.1-3.5) |
| 65-79 | 0.8 (0.4-1.9) | 0.7 (0.1-4.9) | 8.2 (1.9-34.9) | 4.6 (2.5-8.3) |
| 80+ | 1.6 (0.4-7.0) |  | 5.2 (0.6-46.9) | 15.1 (7.0-32.5) |
| Comorbidities | |  |  |  |
| Atrial Fibrillation | 1.1 (0.5-2.5) | 2.3 (0.4-12.3) | 0.7 (0.2-2.9) | 1.3 (0.7-2.2) |
| Cancer | 1.1 (0.7-1.8) | 0.8 (0.4-1.8) | 0.7 (0.3-1.9) | 0.8 (0.5-1.1) |
| Cerebrovascular Disease | 2.6 (1.3-5.1) | 0.9 (0.2-3.1) | 2.2 (0.5-8.9) | 1.4 (0.9-2.1) |
| Coronary Artery Disease | 0.9 (0.5-1.6) | 1.3 (0.3-5.3) | 6.5 (1.9-21.7) | 1.2 (0.8-1.8) |
| Diabetes | 0.6 (0.3-1.2) | 1.3 (0.3-6.4) | 1.2 (0.4-3.5) | 0.8 (0.5-1.2) |
| Heart Failure | 2.3 (1.1-4.9) | 5.7 (1.0-32.4) | 0.7 (0.2-2.3) | 3.4 (1.9-5.9) |
| Hypertension | 1.2 (0.7-2.0) | 2.3 (0.9-6.0) | 0.9 (0.3-2.8) | 1.4 (0.9-2.0) |
| Immunosuppression | 1.3 (0.8-1.9) | 2.4 (1.0-5.3) | 6.0 (2.2-15.8) | 2.8 (1.9-4.1) |
| Kidney Disease | 1.0 (0.6-1.6) | 0.9 (0.3-2.8) | 1.7 (0.5-5.5) | 1.8 (1.2-2.7) |
| Lung Disease | 1.1 (0.8-1.6) | 2.2 (1.3-3.9) | 1.2 (0.6-2.8) | 1.1 (0.8-1.6) |
| Smoker | 1.9 (1.0-3.8) | 2.1 (0.4-10.6) | 0.7 (0.2-2.0) | 1.2 (0.8-1.9) |
| ED Visit | 1.7 (1.1-2.6) | 1.0 (0.4-2.5) | 3.3 (0.8-13.6) | 0.5 (0.3-0.8) |
| Co-infection | 0.5 (0.2-1.4) | 1.9 (0.4-8.1) | 0.8 (0.1-4.4) | 1.2 (0.4-3.9) |

Supplementary Table 1: Odds ratios for multivariable logistic regression covariates for admission

| Supplementary Table 2: Odds ratios for multivariable logistic regression covariates for supplemental oxygen | | | | |
| --- | --- | --- | --- | --- |
|  | RSV | Influenza | Enterovirus/ rhinovirus | SARS-CoV-2 |
| Female | 1.1 (0.6-1.8) | 2.1 (0.9-4.7) | 1.0 (0.7-1.5) | 0.7 (0.5-0.9) |
| Age |  |  |  |  |
| 0-17 | Reference | Reference | Reference | Reference |
| 18-44 | 0.5 (0.1-1.8) | 0.8 (0.3-2.7) | 0.3 (0.2-0.6) | 0.6 (0.3-1.2) |
| 45-64 | 0.5 (0.1-2.9) | 4.6 (1.4-15.8) | 0.6 (0.3-1.2) | 1.3 (0.7-2.7) |
| 65-79 | 1.0 (0.2-5.9) | 6.7 (1.6-27.6) | 0.5 (0.2-1.1) | 2.9 (1.4-5.7) |
| 80+ |  | 5.7 (0.7-44.4) | 0.6 (0.2-1.6) | 5.5 (2.6-11.7) |
| Comorbidities |  |  |  |  |
| Atrial Fibrillation | 1.2 (0.3-4.5) | 0.6 (0.2-2.2) | 0.7 (0.4-1.3) | 1.1 (0.7-1.7) |
| Cancer | 0.7 (0.3-1.6) | 0.6 (0.2-1.6) | 1.1 (0.7-1.7) | 0.8 (0.5-1.2) |
| Cerebrovascular Disease | 1.0 (0.3-3.3) | 2.1 (0.7-6.7) | 1.6 (0.9-2.5) | 1.4 (0.9-2.1) |
| Coronary Artery Disease | 1.4 (0.3-5.7) | 2.7 (0.8-8.7) | 1.3 (0.7-2.3) | 0.9 (0.6-1.5) |
| Diabetes | 1.1 (0.3-3.9) | 0.9 (0.3-2.5) | 0.6 (0.4-1.1) | 1.1 (0.7-1.6) |
| Heart Failure | 2.1 (0.6-7.7) | 1.5 (0.5-4.7) | 1.7 (1.0-2.9) | 2.4 (1.5-3.8) |
| Hypertension | 2.9 (1.1-7.6) | 0.9 (0.2-3.0) | 1.7 (1.1-2.6) | 1.1 (0.7-1.8) |
| Immunosuppression | 1.5 (0.6-3.3) | 3.4 (1.2-9.2) | 0.9 (0.6-1.4) | 2.1 (1.4-3.1) |
| Kidney Disease | 0.7 (0.2-2.2) | 1.1 (0.3-3.3) | 0.9 (0.5-1.4) | 1.7 (1.1-2.5) |
| Lung Disease | 1.9 (1.1-3.4) | 1.7 (0.8-3.9) | 2.1 (1.5-3.0) | 1.9 (1.4-2.8) |
| Smoker | 0.6 (0.2-2.5) | 1.2 (0.4-3.2) | 1.6 (0.9-2.8) | 1.2 (0.8-1.8) |
| ED Visit | 1.5 (0.6-3.8) | 0.9 (0.2-3.1) | 1.2 (0.8-1.8) | 0.9 (0.6-1.5) |
| Co-infection | 2.1 (0.5-7.9) | 0.7 (0.1-5.0) | 0.7 (0.2-2.3) | 0.4 (0.1-1.6) |

Supplementary Table 2: Odds ratios for multivariable logistic regression covariates for supplemental oxygen

| Table S3: RSV Patient Detailed Age groups | |
| --- | --- |
| Age Group | Number (%) |
| <1 | 116 (29.59%) |
| 1 | 68 (17.35%) |
| 2 | 31 (7.91%) |
| 3 | 32 (8.16%) |
| 4 | 23 (5.87%) |
| 5 | 19 (4.85%) |
| 5-17 | 24 (6.15%) |
| 18-44 | 29 (7.45%) |
| 45-64 | 23 (5.92%) |
| 65-79 | 24 (6.15%) |
| 80+ | 3 (0.78%) |

Table S3: RSV Patient Detailed Age groups

|  | 21L (Omicron) | 22A (Omicron) | 22D (Omicron) | 22E (Omicron) | 22F (Omicron) | 23A (Omicron) | 23B (Omicron) | 23C (Omicron) | 23D (Omicron) | 23E (Omicron) | 23F (Omicron) | 23G (Omicron) | 23H (Omicron) | 23I (Omicron) | recombinant |
| --- | --- | --- | --- | --- | --- | --- | --- | --- | --- | --- | --- | --- | --- | --- | --- |
| Jun |  | 1 |  |  | 21 | 28 | 16 |  | 5 |  |  |  |  |  |  |
| Jul |  |  | 1 | 1 | 16 | 25 | 34 |  | 14 | 11 | 14 |  |  |  |  |
| Aug |  |  |  |  | 6 | 34 | 52 | 2 | 41 | 25 | 65 |  |  |  |  |
| Sep |  |  |  |  | 9 | 14 | 23 | 1 | 37 | 24 | 57 |  |  |  | 2 |
| Oct | 1 |  |  |  | 1 | 20 | 18 | 1 | 46 | 6 | 98 |  | 1 |  | 1 |
| Nov |  |  |  |  | 1 | 17 | 16 | 2 | 38 | 6 | 110 | 1 | 3 | 16 | 4 |
| Dec |  |  |  |  |  | 9 | 4 |  | 3 | 2 | 35 | 2 | 2 | 85 | 1 |
| Grand Total | 1 | 1 | 1 | 1 | 54 | 147 | 163 | 6 | 184 | 74 | 379 | 3 | 6 | 101 | 8 |

Table S4. SARS-CoV-2 clades characterized between June- December 2023, JHHS

|  | | | |  |  |  |  |  |  |  |  |  |  |  |  |
| --- | --- | --- | --- | --- | --- | --- | --- | --- | --- | --- | --- | --- | --- | --- | --- |
| Count of lineage | Column Labels |  |  |  |  |  |  |  |  |  |  |  |  |  |  |
| Row Labels | 21L (Omicron) | 22A (Omicron) | 22D (Omicron) | 22E (Omicron) | 22F (Omicron) | 23A (Omicron) | 23B (Omicron) | 23C (Omicron) | 23D (Omicron) | 23E (Omicron) | 23F (Omicron) | 23G (Omicron) | 23H (Omicron) | 23I (Omicron) | recombinant |
| B.1.1.529 |  |  |  |  |  |  |  |  |  | 2 |  |  |  |  |  |
| BA.2.86.1 |  |  |  |  |  |  |  |  |  |  |  |  |  | 5 |  |
| BA.2.86.2 |  |  |  |  |  |  |  |  |  |  |  |  |  | 1 |  |
| BA.2.86.3 |  |  |  |  |  |  |  |  |  |  |  |  |  | 1 |  |
| BA.3 |  |  |  |  |  |  |  |  | 1 |  | 1 |  |  |  |  |
| BA.4.6 |  | 1 |  |  |  |  |  |  |  |  |  |  |  |  |  |
| BQ.1.1.32 |  |  |  | 1 |  |  |  |  |  |  |  |  |  |  |  |
| DV.7.1 |  |  |  |  |  |  |  | 4 |  |  |  |  |  |  |  |
| DV.7.1.1 |  |  |  |  |  |  |  | 1 |  |  |  |  |  |  |  |
| DV.7.1.3 |  |  |  |  |  |  |  | 1 |  |  |  |  |  |  |  |
| EG.1 |  |  |  |  | 1 |  |  |  |  |  |  |  |  |  |  |
| EG.1.4 |  |  |  |  | 1 |  |  |  |  |  |  |  |  |  |  |
| EG.2 |  |  |  |  | 1 |  |  |  |  |  |  |  |  |  |  |
| EG.4 |  |  |  |  | 1 |  |  |  | 1 |  |  |  |  |  |  |
| EG.5 |  |  |  |  |  |  |  |  | 2 |  | 1 |  |  |  |  |
| EG.5.1 |  |  |  |  | 5 |  |  |  |  |  | 43 |  |  |  |  |
| EG.5.1.1 |  |  |  |  | 1 |  |  |  |  |  | 50 |  | 1 |  |  |
| EG.5.1.2 |  |  |  |  |  |  |  |  |  |  | 1 |  |  |  |  |
| EG.5.1.3 |  |  |  |  |  |  |  |  |  |  | 22 |  |  |  |  |
| EG.5.1.4 |  |  |  |  |  |  |  |  |  |  | 25 |  |  |  |  |
| EG.5.1.5 |  |  |  |  |  |  |  |  |  |  | 1 |  |  |  |  |
| EG.5.1.6 |  |  |  |  |  |  |  |  |  |  | 24 |  |  |  |  |
| EG.5.1.8 |  |  |  |  |  |  |  |  |  |  | 2 |  |  |  |  |
| EG.5.1.9 |  |  |  |  |  |  |  |  |  |  | 1 |  |  |  |  |
| EG.5.2 |  |  |  |  |  |  |  |  | 5 |  |  |  |  |  |  |
| EG.5.2.1 |  |  |  |  |  |  |  |  | 1 |  |  |  |  |  |  |
| EG.6.1 |  |  |  |  |  |  |  |  | 9 |  |  |  |  |  |  |
| EG.6.1.1 |  |  |  |  |  |  |  |  | 1 |  |  |  |  |  |  |
| EG.9.1 |  |  |  |  |  |  |  |  | 1 |  |  |  |  |  |  |
| EU.1.1 |  |  |  |  |  | 1 |  |  |  |  |  |  |  |  |  |
| FD.1.1 |  |  |  |  |  | 5 |  |  |  |  |  |  |  |  |  |
| FE.1.1.1 |  |  |  |  | 1 |  |  |  |  |  |  |  |  |  |  |
| FK.1.3 |  |  | 1 |  |  |  |  |  |  |  |  |  |  |  |  |
| FL.1 |  |  |  |  |  |  |  |  | 1 |  |  |  |  |  |  |
| FL.1.5 |  |  |  |  | 1 |  |  |  |  |  |  |  |  |  |  |
| FL.1.5.1 |  |  |  |  | 1 |  |  |  | 73 |  |  |  |  |  |  |
| FL.1.5.2 |  |  |  |  |  |  |  |  | 2 |  |  |  |  |  |  |
| FL.13 |  |  |  |  | 1 |  |  |  |  |  |  |  |  |  |  |
| FL.15 |  |  |  |  |  |  |  |  | 2 |  |  |  |  |  |  |
| FL.15.1.1 |  |  |  |  |  |  |  |  | 1 |  |  |  |  |  |  |
| FL.2 |  |  |  |  |  |  |  |  | 1 |  |  |  |  |  |  |
| FL.2.3 |  |  |  |  |  |  |  |  | 4 |  |  |  |  |  |  |
| FL.2.5 |  |  |  |  |  |  |  |  | 3 |  |  |  |  |  |  |
| FL.25 |  |  |  |  |  |  |  |  | 1 |  |  |  |  |  |  |
| FL.3 |  |  |  |  |  |  |  |  | 1 |  |  |  |  |  |  |
| FL.4 |  |  |  |  | 3 |  |  |  | 1 |  |  |  |  |  |  |
| FL.5 |  |  |  |  |  |  |  |  | 2 |  |  |  |  |  |  |
| FU.1 |  |  |  |  |  |  | 4 |  |  |  |  |  |  |  |  |
| FU.2 |  |  |  |  |  |  | 7 |  |  |  |  |  |  |  |  |
| FU.2.1 |  |  |  |  |  |  | 3 |  |  |  |  |  |  |  |  |
| FY.3 |  |  |  |  | 1 |  |  |  |  |  |  |  |  |  |  |
| FY.3.1 |  |  |  |  | 1 |  |  |  |  |  |  |  |  |  |  |
| FY.5 |  |  |  |  | 1 |  |  |  |  |  |  |  |  |  |  |
| GA.4 |  |  |  |  | 1 |  |  |  |  |  |  |  |  |  |  |
| GD.1 |  |  |  |  |  |  |  |  | 3 |  |  |  |  |  |  |
| GE.1 |  |  |  |  | 2 |  |  |  |  | 9 |  |  |  |  |  |
| GJ.1 |  |  |  |  | 4 |  |  |  |  | 2 |  |  |  |  |  |
| GJ.1.2 |  |  |  |  |  |  |  |  |  | 24 |  |  |  |  |  |
| GK.1 |  |  |  |  |  | 3 |  |  |  |  |  | 1 |  |  |  |
| GK.1.1 |  |  |  |  |  | 12 |  |  |  |  |  |  |  |  |  |
| GK.1.3 |  |  |  |  |  | 1 |  |  |  |  |  |  |  |  | 3 |
| GK.1.4 |  |  |  |  |  | 1 |  |  |  |  |  |  |  |  |  |
| GK.2 |  |  |  |  |  | 5 |  |  |  |  |  | 2 |  |  |  |
| GK.3.1 |  |  |  |  |  | 7 |  |  |  |  |  |  |  |  |  |
| GN.1 |  |  |  |  |  | 3 |  |  |  |  |  |  |  |  |  |
| GN.1.1 |  |  |  |  |  | 1 |  |  |  |  |  |  |  |  |  |
| GS.4.1 |  |  |  |  |  |  |  |  |  | 2 |  |  |  |  |  |
| HF.1 |  |  |  |  |  |  | 1 |  |  |  |  |  |  |  |  |
| HF.1.1 |  |  |  |  |  |  | 1 |  |  |  |  |  |  |  |  |
| HH.1 |  |  |  |  |  |  |  |  |  | 1 |  |  |  |  |  |
| HK.1 |  |  |  |  |  |  |  |  |  |  | 1 |  |  |  |  |
| HK.3 |  |  |  |  |  |  |  |  |  |  | 14 |  | 4 |  |  |
| HK.3.1 |  |  |  |  |  |  |  |  |  |  | 1 |  |  |  |  |
| HK.3.2 |  |  |  |  |  |  |  |  |  |  |  |  | 1 |  |  |
| HK.8 |  |  |  |  |  |  |  |  |  |  | 1 |  |  |  |  |
| HN.1 |  |  |  |  |  |  |  |  | 60 |  |  |  |  |  |  |
| HS.1.1 |  |  |  |  |  | 1 |  |  |  |  |  |  |  |  |  |
| HV.1 |  |  |  |  |  |  |  |  |  |  | 172 |  |  |  |  |
| HZ.1 |  |  |  |  |  | 1 |  |  |  |  |  |  |  |  |  |
| HZ.2 |  |  |  |  |  | 1 |  |  |  |  |  |  |  |  |  |
| JD.1.1 |  |  |  |  |  | 22 |  |  |  |  |  |  |  |  |  |
| JD.1.1.1 |  |  |  |  |  | 6 |  |  |  |  |  |  |  |  |  |
| JD.1.1.3 |  |  |  |  |  | 2 |  |  |  |  |  |  |  |  |  |
| JE.1.1 |  |  |  |  |  |  |  |  |  | 1 |  |  |  |  |  |
| JF.1 |  |  |  |  |  |  | 7 |  |  |  |  |  |  |  |  |
| JG.3 |  |  |  |  |  |  |  |  |  |  | 19 |  |  |  |  |
| JM.2 |  |  |  |  |  |  | 1 |  |  |  |  |  |  |  |  |
| JN.1 | 1 |  |  |  |  |  |  |  |  |  |  |  |  | 93 |  |
| JN.3 |  |  |  |  |  |  |  |  |  |  |  |  |  | 1 |  |
| XBB.1 |  |  |  |  |  |  |  |  | 1 |  |  |  |  |  |  |
| XBB.1.16 |  |  |  |  |  |  | 39 |  |  |  |  |  |  |  |  |
| XBB.1.16.1 |  |  |  |  |  |  | 20 |  |  |  |  |  |  |  |  |
| XBB.1.16.11 |  |  |  |  |  |  | 14 |  |  |  |  |  |  |  |  |
| XBB.1.16.15 |  |  |  |  |  |  | 6 |  |  |  |  |  |  |  |  |
| XBB.1.16.16 |  |  |  |  |  |  | 1 |  |  |  |  |  |  |  |  |
| XBB.1.16.17 |  |  |  |  |  |  |  |  |  |  |  |  |  |  | 3 |
| XBB.1.16.19 |  |  |  |  |  |  | 1 |  |  |  |  |  |  |  |  |
| XBB.1.16.2 |  |  |  |  |  |  | 2 |  |  |  |  |  |  |  |  |
| XBB.1.16.6 |  |  |  |  |  |  | 55 |  |  |  |  |  |  |  |  |
| XBB.1.16.8 |  |  |  |  |  |  | 1 |  |  |  |  |  |  |  |  |
| XBB.1.18.1 |  |  |  |  | 2 |  |  |  |  |  |  |  |  |  |  |
| XBB.1.22 |  |  |  |  | 1 |  |  |  |  |  |  |  |  |  |  |
| XBB.1.4.2 |  |  |  |  | 1 |  |  |  |  |  |  |  |  |  |  |
| XBB.1.41 |  |  |  |  | 4 |  |  |  |  |  |  |  |  |  |  |
| XBB.1.41.1 |  |  |  |  | 3 |  |  |  |  |  |  |  |  |  |  |
| XBB.1.42.1 |  |  |  |  | 2 |  |  |  |  |  |  |  |  |  |  |
| XBB.1.42.2 |  |  |  |  | 2 |  |  |  |  |  |  |  |  |  |  |
| XBB.1.5 |  |  |  |  |  | 29 |  |  |  |  |  |  |  |  |  |
| XBB.1.5.10 |  |  |  |  |  | 7 |  |  |  |  |  |  |  |  |  |
| XBB.1.5.2 |  |  |  |  |  | 1 |  |  |  |  |  |  |  |  |  |
| XBB.1.5.20 |  |  |  |  |  | 1 |  |  |  |  |  |  |  |  |  |
| XBB.1.5.28 |  |  |  |  |  | 3 |  |  |  |  |  |  |  |  |  |
| XBB.1.5.33 |  |  |  |  |  | 2 |  |  |  |  |  |  |  |  |  |
| XBB.1.5.35 |  |  |  |  |  | 1 |  |  |  |  |  |  |  |  |  |
| XBB.1.5.37 |  |  |  |  |  | 1 |  |  |  |  |  |  |  |  |  |
| XBB.1.5.39 |  |  |  |  |  | 2 |  |  |  |  |  |  |  |  |  |
| XBB.1.5.4 |  |  |  |  |  | 1 |  |  |  |  |  |  |  |  |  |
| XBB.1.5.49 |  |  |  |  |  | 3 |  |  |  |  |  |  |  |  |  |
| XBB.1.5.50 |  |  |  |  |  | 1 |  |  |  |  |  |  |  |  |  |
| XBB.1.5.51 |  |  |  |  |  | 2 |  |  |  |  |  |  |  |  |  |
| XBB.1.5.57 |  |  |  |  |  | 1 |  |  |  |  |  |  |  |  |  |
| XBB.1.5.59 |  |  |  |  |  | 1 |  |  |  |  |  |  |  |  |  |
| XBB.1.5.66 |  |  |  |  |  | 1 |  |  |  |  |  |  |  |  |  |
| XBB.1.5.70 |  |  |  |  |  | 3 |  |  |  |  |  |  |  |  |  |
| XBB.1.5.72 |  |  |  |  |  | 11 |  |  |  |  |  |  |  |  |  |
| XBB.1.5.73 |  |  |  |  |  | 2 |  |  |  |  |  |  |  |  |  |
| XBB.1.5.77 |  |  |  |  |  | 1 |  |  |  |  |  |  |  |  |  |
| XBB.1.5.78 |  |  |  |  |  | 1 |  |  |  |  |  |  |  |  |  |
| XBB.1.9.1 |  |  |  |  | 1 |  |  |  | 2 |  |  |  |  |  |  |
| XBB.1.9.2 |  |  |  |  | 4 |  |  |  | 5 |  |  |  |  |  |  |
| XBB.2.3 |  |  |  |  | 3 |  |  |  |  | 9 |  |  |  |  |  |
| XBB.2.3.11 |  |  |  |  |  |  |  |  |  | 4 |  |  |  |  |  |
| XBB.2.3.13 |  |  |  |  |  |  |  |  |  | 1 |  |  |  |  |  |
| XBB.2.3.2 |  |  |  |  | 3 |  |  |  |  | 5 |  |  |  |  |  |
| XBB.2.3.3 |  |  |  |  | 1 |  |  |  |  | 2 |  |  |  |  |  |
| XBB.2.3.8 |  |  |  |  |  |  |  |  |  | 12 |  |  |  |  |  |
| XBC.1.6.2 |  |  |  |  |  |  |  |  |  |  |  |  |  |  | 1 |
| XCH |  |  |  |  |  |  |  |  |  |  |  |  |  |  | 1 |
| Grand Total | 1 | 1 | 1 | 1 | 54 | 147 | 163 | 6 | 184 | 74 | 379 | 3 | 6 | 101 | 8 |

Table S5. SARS-CoV-2 lineages distribution by clade**.**

|  | Negative | Positive | Grand Total |
| --- | --- | --- | --- |
| 21L (Omicron) |  | 1 | 1 |
| 22E (Omicron) | 1 |  | 1 |
| 22F (Omicron) | 7 | 10 | 17 |
| 23A (Omicron) | 43 | 33 | 76 |
| 23B (Omicron) | 29 | 44 | 73 |
| 23C (Omicron) | 3 | 1 | 4 |
| 23D (Omicron) | 21 | 62 | 83 |
| 23E (Omicron) | 14 | 16 | 30 |
| 23F (Omicron) | 42 | 123 | 165 |
| 23H (Omicron) | 1 | 1 | 2 |
| 23I (Omicron) | 13 | 21 | 34 |
| recombinant | 1 | 7 | 8 |
| Grand Total | 175 | 319 | 494 |

Table S6. SARS-CoV-2 positive samples used for cell culture.

|  | **Count of clade** |
| --- | --- |
| **H1N1** | **246** |
| 6B.1A.5a.2a | 102 |
| 6B.1A.5a.2a.1 | 144 |
| **H3N2** | **46** |
| 3C.2a1b.2a | 1 |
| 3C.2a1b.2a.2a.1b | 1 |
| 3C.2a1b.2a.2a.3a.1 | 38 |
| 3C.2a1b.2a.2b | 6 |
| **Influenza B** | **38** |
| V1A.3a.2 | 38 |
| **Grand Total** | **330** |

Table S7. Influenza clades identified in our study.

|  | **Count of Type** |
| --- | --- |
| **Enterovirus A** | **11** |
| CV-A2 | 3 |
| CV-A5 | 2 |
| CV-A6 | 6 |
| **Enterovirus B** | **18** |
| Could not assign | 1 |
| CV-A9 | 2 |
| CV-B4 | 5 |
| CV-B5 | 2 |
| E18 | 1 |
| E-18 | 5 |
| E-5 | 1 |
| E-7 | 1 |
| **Enterovirus C** | **1** |
| E C105 | 1 |
| **Enterovirus D** | **4** |
| EV-D68 | 4 |
| **Rhinovirus A** | **260** |
| Could not assign | 3 |
| HRV-A1 | 1 |
| HRV-A10 | 15 |
| HRV-A101 | 4 |
| HRV-A103 | 1 |
| HRV-A106 | 2 |
| HRV-A12 | 11 |
| HRV-A15 | 3 |
| HRV-A18 | 1 |
| HRV-A19 | 1 |
| HRV-A2 | 12 |
| HRV-A20 | 8 |
| HRV-A21 | 1 |
| HRV-A22 | 5 |
| HRV-A25 | 2 |
| HRV-A28 | 2 |
| HRV-A29 | 11 |
| HRV-A30 | 8 |
| HRV-A31 | 36 |
| HRV-A32 | 1 |
| HRV-A33 | 1 |
| HRV-A34 | 5 |
| HRV-A38 | 3 |
| HRV-A40 | 2 |
| HRV-A47 | 6 |
| HRV-A49 | 15 |
| HRV-A51 | 5 |
| HRV-A53 | 2 |
| HRV-A54 | 2 |
| HRV-A55 | 2 |
| HRV-A56 | 1 |
| HRV-A57 | 1 |
| HRV-A60 | 3 |
| HRV-A61 | 1 |
| HRV-A63 | 2 |
| HRV-A66 | 1 |
| HRV-A68 | 3 |
| HRV-A7 | 2 |
| HRV-A71 | 2 |
| HRV-A73 | 18 |
| HRV-A75 | 8 |
| HRV-A76 | 4 |
| HRV-A77 | 7 |
| HRV-A78 | 1 |
| HRV-A80 | 1 |
| HRV-A81 | 8 |
| HRV-A82 | 6 |
| HRV-A89 | 2 |
| HRV-A9 | 9 |
| HRV-A98 | 9 |
| **Rhinovirus B** | **135** |
| HRV-B101 | 3 |
| HRV-B27 | 2 |
| HRV-B3 | 1 |
| HRV-B37 | 1 |
| HRV-B4 | 1 |
| HRV-B42 | 3 |
| HRV-B48 | 1 |
| HRV-B52 | 24 |
| HRV-B6 | 9 |
| HRV-B69 | 23 |
| HRV-B70 | 4 |
| HRV-B84 | 1 |
| HRV-B92 | 62 |
| **Rhinovirus C** | **248** |
| Could not assign | 7 |
| HRV-A13 | 1 |
| HRV-C1 | 22 |
| HRV-C10 | 2 |
| HRV-C11 | 23 |
| HRV-C12 | 4 |
| HRV-C13 | 4 |
| HRV-C15 | 10 |
| HRV-C17 | 24 |
| HRV-C2 | 4 |
| HRV-C20 | 3 |
| HRV-C21 | 1 |
| HRV-C25 | 1 |
| HRV-C26 | 5 |
| HRV-C28 | 2 |
| HRV-C3 | 1 |
| HRV-C30 | 3 |
| HRV-C32 | 2 |
| HRV-C33 | 1 |
| HRV-C35 | 22 |
| HRV-C36 | 7 |
| HRV-C37 | 1 |
| HRV-C40 | 22 |
| HRV-C41 | 8 |
| HRV-C42 | 6 |
| HRV-C43 | 19 |
| HRV-C45 | 1 |
| HRV-C46 | 2 |
| HRV-C51 | 1 |
| HRV-C56 | 6 |
| HRV-C6 | 16 |
| HRV-C7 | 6 |
| HRV-C8 | 11 |
| **Grand Total** | **677** |

Table S8. Enterovirus/ rhinovirus species and types identified in our study.

|  | **Count of clade** |
| --- | --- |
| **RSV A** | **79** |
| GA2.3.5 | 73 |
| GA2.3.5 / Gb5.0.5a | 4 |
| Could not assign | 2 |
| **RSV A** | **11** |
| GA2.3.5 | 7 |
| Could not assign | 4 |
| **RSV A/B** | **10** |
| GA2.3.5 | 5 |
| GA2.3.5 / Gb5.0.5a | 3 |
| GB5.0.5a / GA2.3.5 | 1 |
| Could not assign | 1 |
| **RSV B** | **292** |
| GB5.0.5a | 286 |
| Could not assign | 6 |
| **Grand Total** | **392** |

Table S9. RSV clades identified in our study.

**Supplementary Figure Legends.**

Figure S1. Positivity rates of respiratory virus testing at JHHS from January 2020 to December 2023. HPIV, human parainfluenza virus, HCoV, human endemic coronaviruses.
